# Clinical, Pathological, and Molecular Characteristics in Colorectal Cancer

**DOI:** 10.3390/cancers14235958

**Published:** 2022-12-02

**Authors:** Stéphane Dedieu, Olivier Bouché

**Affiliations:** 1 CNRS UMR 7369 MEDyC, Matrice Extracellulaire et Dynamique Cellulaire, 51100 Reims, France; 2 Campus Moulin de la Housse, UFR Sciences Exactes et Naturelles, Université de Reims Champagne-Ardenne (URCA), 51100 Reims, France; 3 BioSpecT, UFR Médecine et Pharmacie, Université Reims Champagne-Ardenne (URCA), 51100 Reims, France; 4 Digestive Oncology and Hepato-Gastroenterology Department, CHU Reims, 51100 Reims, France

Colorectal cancer (CRC) is the third most diagnosed cancer worldwide, and the second leading cause of death in patients with cancer. Lifestyle, diet factors, obesity, family history, or even pre-existing inflammatory diseases lead to increased risks of developing this heterogeneous malignant disease.

Carcinogenesis steps have commonly involved the growth and expansion of adenomatous polyps from normal colorectal epithelium to adenoma through a multistep process of several years. Many signaling pathways are altered during this transformation course that involves, certainly, hereditary factors, but mainly somatic sporadic mutations affecting both tumor suppressor genes and oncogenes (*TP53*, *APC*, and *KRAS* from the most common recurrent somatic mutations). Chromosomal instability, CpG island methylation phenotype, and microsatellite instability (MSI) frequency are the three main routes leading to tumor transformation and progression.

The most common tumor–node–metastasis (TNM) staging system driving CRC patient treatment is not completely satisfactory because patients with similar histopathology may have various therapeutic responses and relapse frequencies due to differential genetic and epigenetic profiles. New biomarkers such as Immunoscore ^®^, gene signatures, and postoperative circulating tumor DNA or extracellular vesicles are promising tools used to identify patients with a high risk of recurrence after primary tumor resection.

Patient prognosis has improved over the past few decades in developed countries, due to an improved health path and better awareness of the population to diagnosis, earlier and regular screening, and access to more extensive surgery and more effective targeted therapies. However, the 5-year survival rate of patients with stage IV remains under 10%. Drug development efforts are therefore mainly focused on patients with stage IV metastatic CRC (mCRC). Although surgery is the primary curative treatment of early-stage patients and resectable metastasis, current treatments for unresectable mCRC involve cytotoxic chemotherapies and targeted therapies, either alone or as a combination treatment. Approved targeted therapy includes angiogenesis inhibitors (bevacizumab, aflibercept, and regorafenib), anti-EGFR monoclonal antibodies (cetuximab and panitumumab) in RAS wild-type tumors, and tyrosine kinase BRAF/MEK inhibitors (binimetinib and encorafenib) in BRAF-mutated tumors.

The emergence and success of immunotherapies in other indications could change the game. Despite a wide variety of immunotherapy approaches in early-phase clinical trials, clinical benefits are currently limited to mCRC patients with a microsatellite instability-high (MSI-H) or mismatch repair-deficient (dMMR) profile, representing approximately 15% of patients. However, recent data also suggest an upfront role for immunotherapy in resectable early-stage MSI-H/dMMR CRC.

A better understanding of the specific clinical and/or molecular features in CRC should therefore improve patient stratification and follow-up, together with treatment algorithms, especially when there is a significant unmet medical need. This will require the identification of new biomarkers and therapeutic targets to overcome current barriers and limitations.

Through original articles, this Special Issue provides novel findings on biomarkers of prognostic relevance. Interestingly, PD-L1 expression succeeds in discriminating patients with differential prognosis in the consensus molecular subtype (CMS)2/3 [1], considering overall and disease-free survival [2]. Regarding metastasis-driving protein biomarkers, PD-L1 as well as α2β1 integrin, CD44v6, IGF-1R, and EGF-R exhibit distinct expression patterns depending on the metastatic organ site [3]. Selective pharmacological targeting based on these molecular signatures could thus faciliate the differential treatment of distant metastases according to their specific metastatic locations.

The expression of discoidin domain receptors (DDRs), i.e., collagen receptors with tyrosine kinase activity, was furthermore investigated in a large cohort of CRC patients [4]. DDRs were found highly expressed in colon adenocarcinoma and associated with a molecular profile that could be integrated within the CMS4 group. While its role as a prognosis marker remains uncertain, DDR expression was found to be associated with shorter event-free survival in CRC patients. 

By developing a comprehensive liquid biopsy profile in mCRC patients, Sachin Narayan et al. [5] have also highlighted and characterized heterogeneous populations of oncosomes and CTCs. Although studies including larger numbers of patients are needed for clinical validation, this work supports the predictive benefit of liquid biopsy in the follow-up of mCRC. In a complementary view, the study by Izabela Papiewska-Pająk et al. [6] emphasized the importance of the miRNA content of extracellular vesicle released by CRC for supporting tumor progression, which may be useful as a biomarker indicating the stage of CRC.

In addition, an original study analyzed the contribution of the genetic component to CRC risk in the Basque population with a specific genetic history [7], while another assessed the importance of routine immunohistochemistry screening for MMR status in CRC patients in the identification of Lynch syndrome patients [8].

Considering the prognosis heterogeneity of CRC patients with stage II or III, David Viñal and colleagues [9] proposed a simple score using three clinico-pathological parameters available in routine clinical practice (T4, N2, and high tumor budding) to stratify the recurrence risk and patient prognosis.

In a precision medicine approach, the potential of HER-2 targeting in mCRC was reviewed [10], and a comprehensive update was provided on the CXCL12/CXCR4/CXCR7 axis, including pharmacological perspectives [11]. Finally, the review by Rami Rhaiem and colleagues [12] discussed data on the role of RAS mutational status in tailoring the surgical and/or thermal ablation approach of colorectal liver metastases.

Thanks to advances in molecular biology identifying theranostic biomarkers from tissues but also liquid biopsies, the future lies in increasingly personalized management for the therapeutic choice and monitoring of patients with mCRC.
